# Optical bistability in a nonlinear-shell-coated metallic nanoparticle

**DOI:** 10.1038/srep21741

**Published:** 2016-02-24

**Authors:** Hongli Chen, Youming Zhang, Baile Zhang, Lei Gao

**Affiliations:** 1 College of Physics, Optoelectronics and Energy of Soochow University, & Collaborative Innovation Center of Suzhou Nano Science and Technology, Soochow University, Suzhou 215006, China; 2 Division of Physics and Applied Physics, School of Physical and Mathematical Sciences, Nanyang Technological University, Singapore 637371, Singapore; 3 Centre for Disruptive Photonic Technologies, Nanyang Technological University, Singapore 637371, Singapore; 4 Jiangsu Key Laboratory of Thin Films, Soochow University, Suzhou 215006, China

## Abstract

We provide a self-consistent mean field approximation in the framework of Mie scattering theory to study the optical bistability of a metallic nanoparticle coated with a nonlinear shell. We demonstrate that the nanoparticle coated with a weakly nonlinear shell exhibits optical bistability in a broad range of incident optical intensity. This optical bistability critically relies on the geometry of the shell-coated nanoparticle, especially the fractional volume of the metallic core. The incident wavelength can also affect the optical bistability. Through an optimization-like process, we find a design with broader bistable region and lower threshold field by adjusting the size of the nonlinear shell, the fractional volume of the metallic core, and the incident wavelength. These results may find potential applications in optical bistable devices such as all-optical switches, optical transistors and optical memories.

Optical bistability has attracted remarkable attention in recent years because of its promising applications in logic functions^1^, metamaterials^2^,^3^,^4^,^5^, all-optical switching^6^ and low power lasing^7^. An optical bistable system has two stable transmission states to switch on and off, depending upon the history of input signal^8^,^9^,^10^. As a conventional type of nonlinear materials, Kerr materials are widely studied in optical bistability. In view of its weak nonlinearity, sophisticated structural design is needed in Kerr nonlinear devices, aiming for faster switching speed and broader range of operation for incident intensity^11^.

In this reports, we study the optical bistability of a metallic nanoparticle coated with a nonlinear shell based on a self-consistent mean field approximation in the framework of Mie scattering theory. We decompose the scattered fields of the coated nanosphere into spherical waves with Debye potentials in order to establish the relationship between the local field of the shell and the incident field. The self-consistent mean field approximation is then adopted to study the optical bistability. Our results match well with the previous quasi-static solution of the Laplace equations at a deep-subwavelength scale^12^. On the other hand, our full-wave solutions are valid in a more strict sense, and thus are more suitable for structural design of optical bistable devices, in which various parameters need to be varied in a wide parameters space.

## Results

### Theoretical development

**Figure 1:**
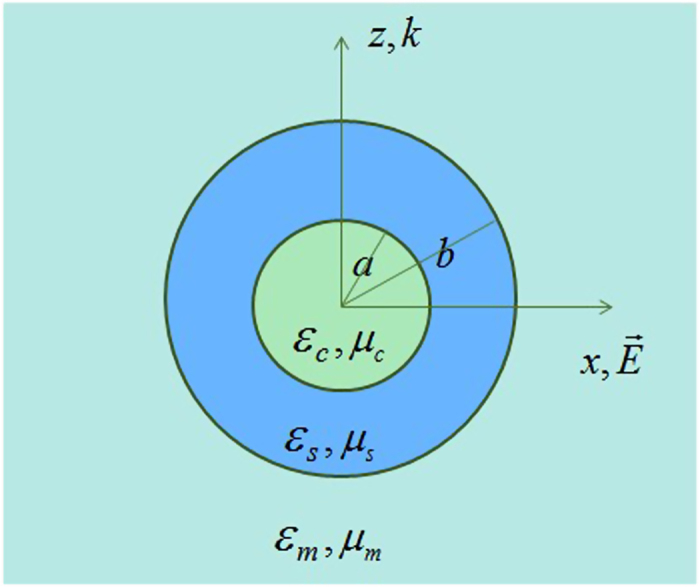
Geometry of scattering of a plane wave by a coated sphere. The radius of the core is *a* and the outer radius is *b*. The incident plane wave is polarization along the 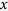-direction and propagates along 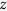-direction. The relative permittivity and permeability of the core (the shell) are 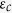 and 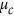 (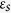 and 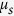). The surrounding medium has a relative permittivity 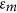 and a relative permeability 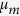.

We first consider electromagnetic wave scattering from the coated metallic nanoparticle. The coated particle, as shown in Fig. 1, has a metallic core with radius *a* and a shell with outer radius *b*. The relative permittivity and permeability of the core (the shell) are 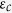 and 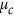 (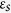 and 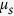). The surrounding medium has a relative permittivity 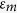 and a relative permeability 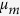. We assume that the incident plane wave propagates in 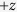 direction, with electric field polarized in *x* direction:


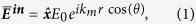


where


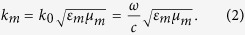


In time-harmonic cases, Maxwell’s equations in the core, shell and surrounding media can be written as 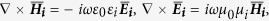 where 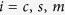, denote ‘core’, ‘shell’ and ‘surrounding medium’, respectively. When all media are linear, fields can be expressed with Debye potentials. By matching the boundary conditions at *r* = *a* and *r* = *b*, the scattered field in the surrounding medium and the fields in the core and shell can all be solved for both transverse-electric (TE) and transverse-magnetic (TM) waves (see details in **Methods**). Since the nanoparticle under consideration is much smaller than the wavelength, the 1^st^ order TM wave is generally sufficient for numerical calculation. More orders can be included if more accurate results are required.

Now, we consider the case in which the coated shell is a Kerr material with weak nonlinearity. The electric displacement 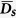 and the electric field 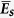 in the shell can be written as:


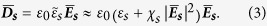


Here 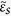 is the nonlinear permittivity of the shell, which is related to the linear permittivity 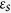, the nonlinear susceptibility 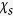 and the local electric field intensity 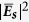 of the coated shell. To solve the field in the nonlinear shell, we adopt self-consistent mean field approximation^12^,^13^,^14^. The nonlinearity of the shell is very weak, meaning that the linear part 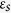 is much larger than the nonlinear part *χ*_*s* _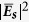. Thus, the nonlinear permittivity of the shell can be expressed as:


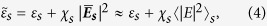


where 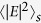 corresponds to the average of the field intensity in a linear shell. It can be calculated as:


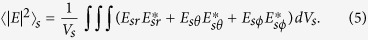


Following Refs 15 and 16, we obtain,


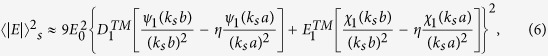


where 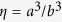 is the fractional volume of the metallic core, 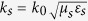. 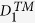 and 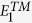 are the expansion coefficients of Debye potentials for TM waves. Detailed expressions can be found in **Method**. After replacing the linear permittivity 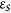 in Eq. (6) with the field-dependent nonlinear permittivity 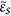 in Eq. (4), we can obtain a bistable relation between electric field intensity of the incident wave 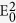 and the average electric field intensity in the nonlinear shell 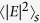.

The optical bistable response of a coated sphere has been studied previously using quasi-static approximation^12^ which shows the relation between the local field average in the shell and the external applied field as


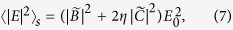


where


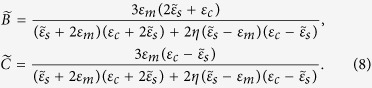


Our results of full-wave solutions will be compared with previous results using quasi-static approximation^12^ in the next section.

### Numerical calculations

In our calculations, we set the linear permittivity of the shell as 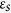 = 2.2 and the metallic core with a permittivity following Drude model^17^


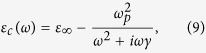


where 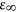 = 3.7, 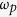 = 8.9 eV and 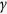 = 0.021 eV. The surrounding medium has a permittivity of 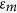 = 1. The relative permeability of the metallic core 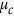, the shell 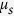 and the surrounding medium 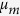 are all set as unit.

**Figure 2:**
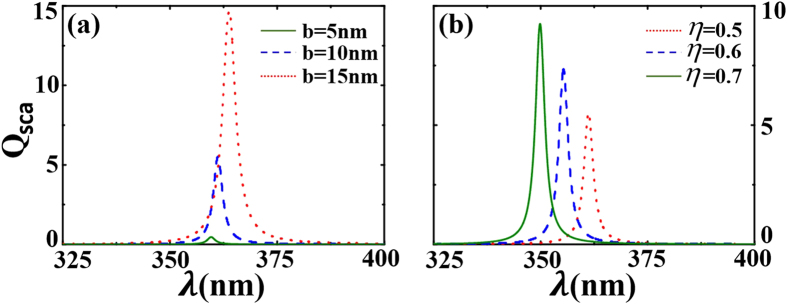
The scattering efficiency vs wavelength diagrams when (**a**) 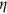 is fixed at 0.5 and (**b**) 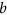 is fixed at 10 nm. The permittivity of the media 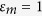.

**Figure 3:**
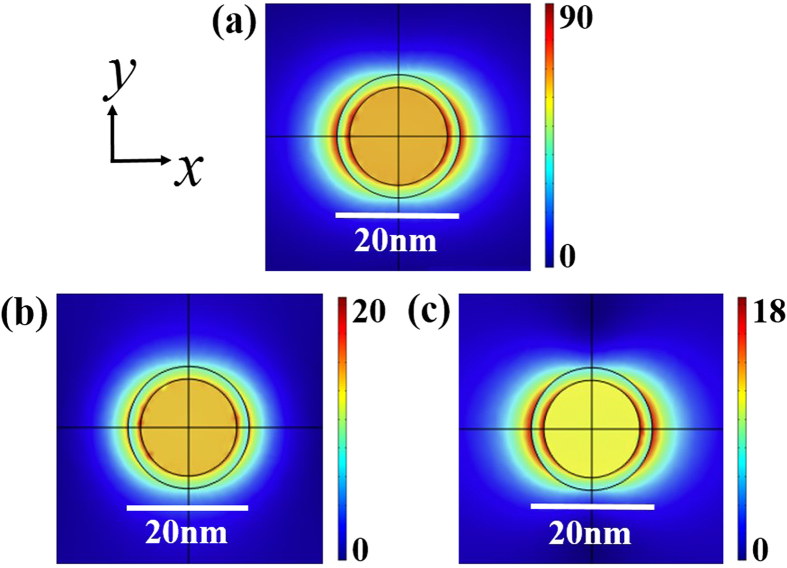
Distributions of the electric field for (**a**) 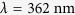, (**b**) 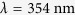 and (**c**) 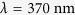. The other parameters are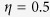 and 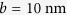.

In Fig. 2, we show the scattering efficiency of the linear coated nanoparticle, which is defined as 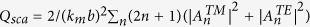 (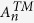 and 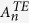 are scattering coefficients in **Method)**. It can be seen that resonance enhancement can be found when the size of the coated nanoparticle *b*, or the fractional volume of the core 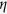, changes. As shown in Fig. 2(a), the enhanced resonance is red-shifted when the size of the coated nanoparticle increases (with a fixed *η*). In Fig. 2(b), the enhanced resonance is blue-shifted when 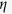
increases (with a fixed size *b*). The near field properties of the linear coated nanoparticle at different wavelengths are shown in Fig. 3. The excited surface plasmons bring out enhanced field at the resonant wavelength in the shell, justifying the consideration of nonlinearity in the shell.

**Figure 4:**
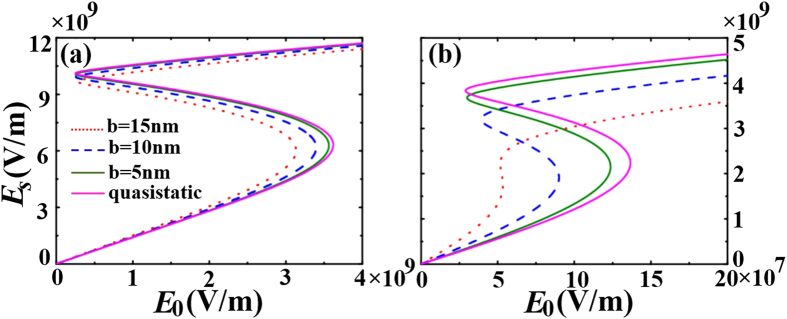
The average local field *E*_*s*_ as a function of the incident field *E*_0_ for various sizes of the sphere. The relevant parameters are (**a**) 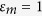 (**b**) 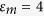. And the core-shell-volumes ratio 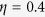 and the incident wavelength 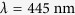 (the resonance wavelength for 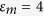, 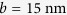 and 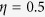).

Next, we introduce weak nonlinearity into the shell. The nonlinear relative permittivity of the shell is set as^18^ 
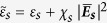
, where 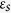 = 2.2, 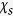 = 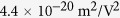. The relations between the electric field amplitude of the incident wave 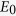 and the average electric field amplitude in the nonlinear shell 
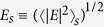
 for different shell sizes (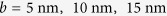) and the relation calculated with quasi-static approximation in different surrounding media (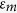 = 1 and 4) are shown in Fig. 4. The fractional volume of the core is fixed as 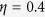. Bistable responses can be clearly observed. Take 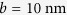 as an example. The electric field in the shell first increases as the incident field increases from zero. When the incident field amplitude reaches the switching-up threshold field 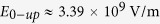 (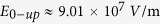) for 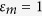 (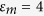), the electric field amplitude in the shell will discontinuously jump to the upper stable branch. If the incident field is decreased back from a large value to zero, the electric field in the shell will first decrease continuously, and then jump down to the lower stable branch when the incident field amplitude reaches the switching-down threshold field 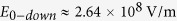 (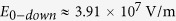) for 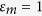 (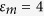). Comparing the relation curves for different shell sizes and the curve calculated with quasi-static approximation, we find that the bistable region (the difference value between the switching-up threshold and the switching-down threshold) in terms of the range of input power decreases and the switching-up threshold become lower as the size of the sphere increases. The results for very small nanoparticles match well with those of quasi-static approximation, but some deviation starts to occur when the size of the nanoparticle increases.

**Figure 5:**
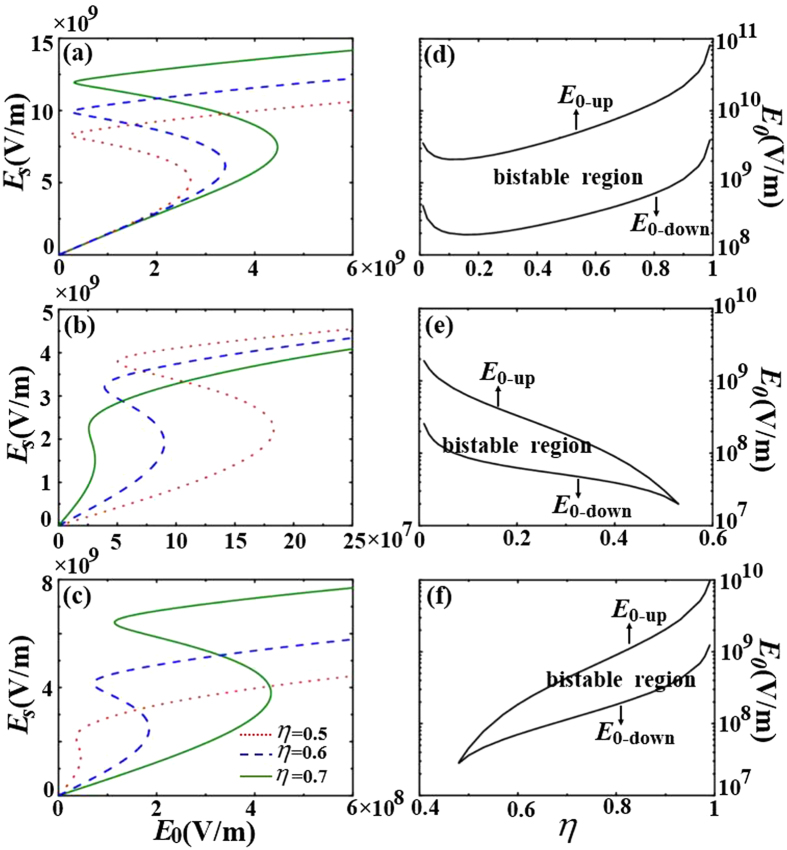
The average local field *E*_*s*_ as a function of the incident field *E*_0_ for various sizes of the sphere. The size of the coated sphere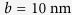, the incident wavelength 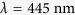 and the permittivity for the media (**a**) 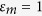 (**b**) 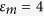. In (**c**), 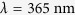 and 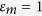. The switching-up and switching-down threshold fields as a function of 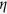 for (**d**) 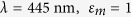 (**e**) 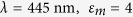 and (**f**) 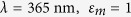.

Then, we study the influence of the fractional volume of metallic core 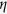 on the bistability. We fix the size of the shell 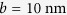. In Fig. 5(a,d) for 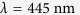
and 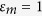, bistable responses can be clearly seen over the whole range of the fractional volume of metallic core 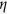. However, when the surrounding medium is changed to possess 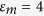, as shown in Fig. 5(b,e), the bistability disappears when the fractional volume of metallic core 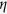 goes beyond a critical value 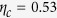. If we keep 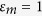 and lower the wavelength to 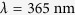, as shown in Fig. 5(c,f), we find that the bistability disappears when 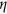 decreases below a critical value 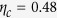.

**Figure 6:**
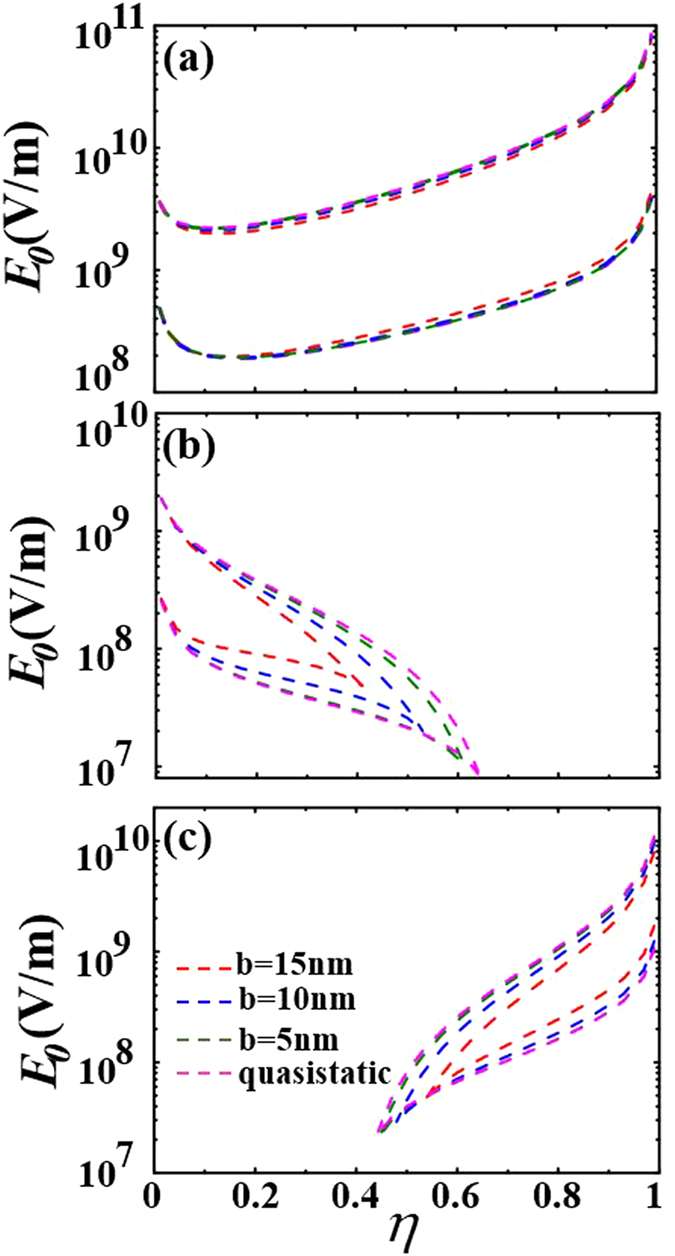
The switching-up and switching-down threshold fields as a function of 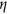 for (**a**) 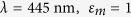 (**b**) 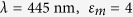 and (**c**) 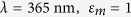.

The shell size can also affect the bistable behavior. As shown in Fig. 6(a), the switching-up and switching-down threshold fields are almost unchanged as the shell size increases for 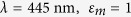. However, as shown in Fig. 6(b), the maximum critical fractional volume of metallic core 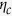 for 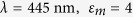 decreases as the shell size increases. As shown in Fig. 6(c), the minimum critical fractional volume of metallic core 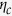 for 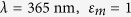 increases as the shell size increases. As a consequence, the bistable region becomes broader as the shell size decreases.

**Figure 7:**
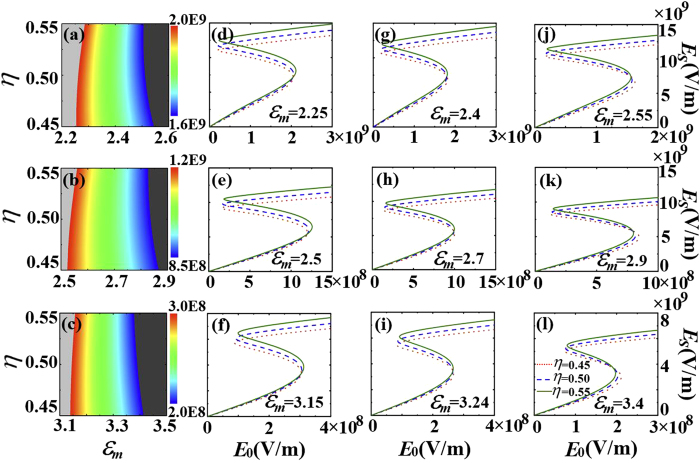
(**a–c**) Contour plots of switching-up threshold 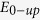 as function of 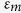 and 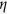. The dark gray region represents that the values are less than the color scale, while those in the light gray region surpass the color scale. (**d–l**)The average local field 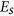 as a function of the incident field 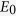. The size of the sphere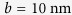 and the incident wavelength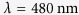. The linear relative permittivity 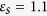 for the first row of panels, 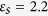 for the second row of panels, and 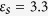 for the third row of panels.

In Fig. 5(f), we find that the switching-up threshold field increases and the bistable region widens as the fractional volume 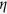 increases when the permittivity of the surrounding medium is 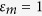. On the contrary, in Fig. 5(e), the switching-up threshold field decreases and the bistable region becomes narrow as the fractional volume 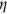 increases when permittivity of the surrounding medium is 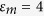. We thus speculate that there is critical surrounding permittivity 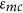 for such a transition. We plot the electric field amplitude in the shell versus the incident field amplitude for different linear part of the shell permittivity (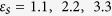). As can be seen in Fig. 7(a–c), the contour plots of 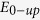 are nearly trapezoids. Thus, at the critical surrounding permittivity, the contour of the switching-up threshold fields should be vertical. In Fig. 7(a), the critical surrounding permittivity nearly equals 2.4 when 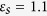. When the surrounding permittivity 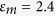 [Fig. 7(g)], the switching-up threshold field is nearly unchanged with increasing the fractional volume 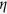. When the surrounding permittivity is less than the critical one, as shown in Fig. 7 (d), the switching-up threshold field increases with increasing the fractional volume 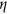. In Fig. 7(j), the switching-up threshold field decreases with increasing the fractional volume 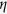 because the surrounding permittivity is more than the critical one. These properties also apply to the cases when 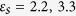, as shown in the lower two rows of panels in Fig. 7.

**Figure 8:**
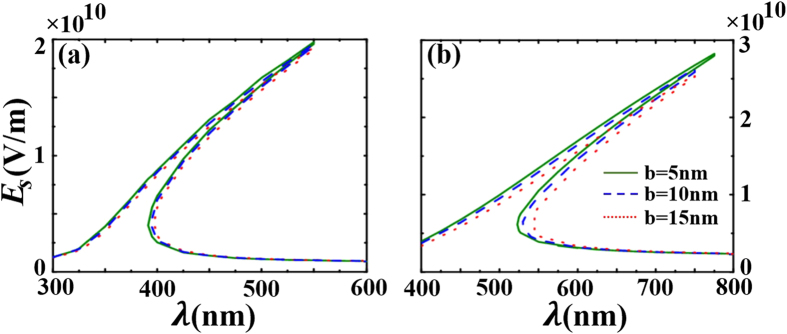
The average local field 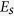 versus wavelength for different sizes and the permittivity of the surrounding medium (**a**) 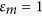 (**b**) 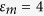.

At last, we fix the incident field amplitude 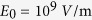, and study how the shell size affects the relationship between the average local field in the shell and the incident wavelength. It can be seen from Fig. 8 that, for 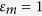 and 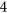, the switching-up wavelength at the fixed input power blue-shifts when the shell sizes increase, while the switching-down wavelength at the fixed input power red-shifts when the shell sizes increase. In Fig. 8(b), when the wavelength reaches about 775 nm (the switching-up wavelength for 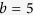 nm and the switching-up wavelength is about 750 nm for 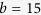 nm), the electric field amplitude in the shell will discontinuously jump to the lower stable branch. However, if one decreases the wavelength to about 520 nm (the switching-down wavelength for 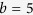 nm and the switching-down wavelength is about 540 nm for 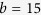 nm), the electric field amplitude in the shell will discontinuously jump up to the upper stable branch.

## Conclusions

In this reports, we adopt self-consistent mean field approximation within the framework of Mie scattering theory to study the optical bistability of a nonlinear coated metallic nanoparticle. Introducing weak nonlinearity to the shell, we demonstrate numerically that the metallic nanoparticle coated with a nonlinear shell has broad bistable region. We study the effect of the size of the coated spheres, the fractional volume of the metallic core, the permittivity of the surrounding medium, as well as the incident wavelength on the hysteresis loops and the switching-up and switching-down threshold fields. While our results match well with the previous quasi-static results, our full-wave solutions based on Mie scattering are valid in a much wide range of parameters, and thus are more suitable for design of optical bistable devices in optimization.

## Methods

### Debye potentials

We express the Debye potentials of the incident fields^19^,


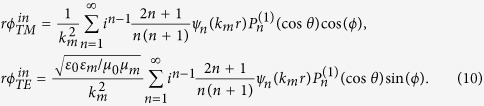


The Debye potentials of the scattering wave are


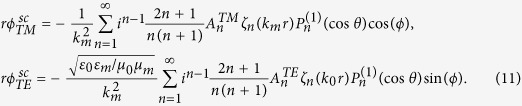


Then, in the shell, the Debye potentials are


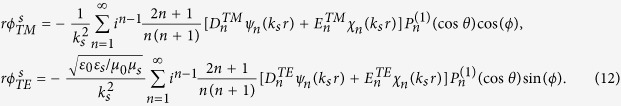


In the core, they should be written as


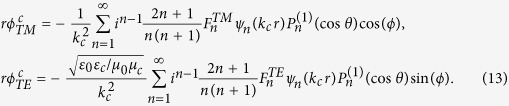


where 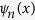, 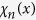 and 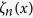 are the Ricatti-Bessel functions and they can be defined by 
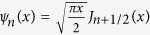
, 
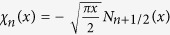
 and 
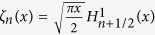
. Here, 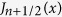, 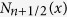 and 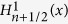 are the Bessel functions , Neumann functions and the first-kind Hankel functions. 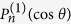 are the associated Legendre polynomials. In addition, we denote 
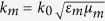
, 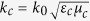 and 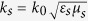.

### Boundary conditions and expansion coefficients of Debye potentials

To solve the coefficients, we apply the boundary conditions on 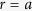 and 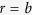. They are


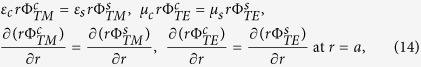


and


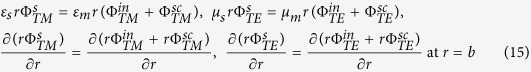


Substituting the boundary conditions into the Debye potentials, we can obtain the coefficients as follows,


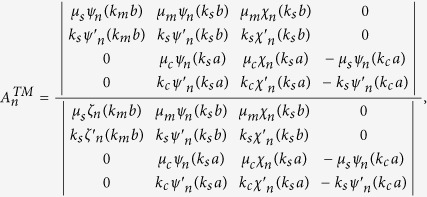



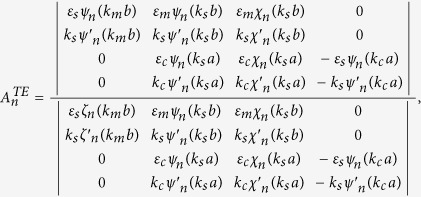



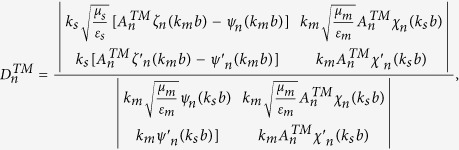



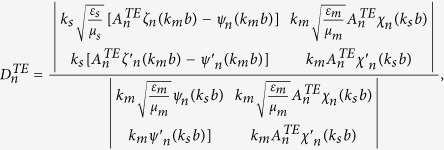



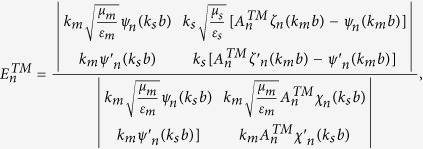



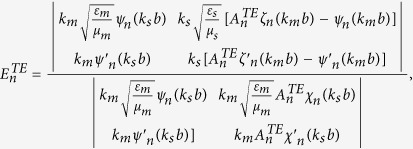



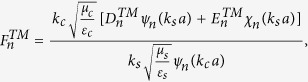


and


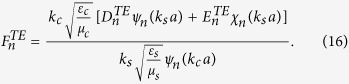


## Additional Information

**How to cite this article**: Chen, H. *et al*. Optical bistability in a nonlinear-shell-coated metallic nanoparticle. *Sci. Rep.* **6**, 21741; doi: 10.1038/srep21741 (2016).
